# Mechanisms of DNA opening revealed in AAA+ transcription complex structures

**DOI:** 10.1126/sciadv.add3479

**Published:** 2022-12-21

**Authors:** Fuzhou Ye, Forson Gao, Xiaojiao Liu, Martin Buck, Xiaodong Zhang

**Affiliations:** ^1^ Section of Structural and Synthetic Biology, Department of Infectious Disease, Faculty of Medicine, Imperial College London, South Kensington SW7 2AZ, UK.; ^2^ Department of Life Sciences, Imperial College London, South Kensington SW7 2AZ, UK.

## Abstract

Gene transcription is carried out by RNA polymerase (RNAP) and requires the conversion of the initial closed promoter complex, where DNA is double stranded, to a transcription-competent open promoter complex, where DNA is opened up. In bacteria, RNAP relies on σ factors for its promoter specificities. Using a special form of sigma factor (σ^54^), which forms a stable closed complex and requires its activator that belongs to the AAA+ ATPases (ATPases associated with diverse cellular activities), we obtained cryo–electron microscopy structures of transcription initiation complexes that reveal a previously unidentified process of DNA melting opening. The σ^54^ amino terminus threads through the locally opened up DNA and then becomes enclosed by the AAA+ hexameric ring in the activator-bound intermediate complex. Our structures suggest how ATP hydrolysis by the AAA+ activator could remove the σ^54^ inhibition while helping to open up DNA, using σ^54^ amino-terminal peptide as a pry bar.

## Teaser

cryoEM structures reveal DNA opening by a protein peptide threading through the DNA double helix in transcription initiation.

## INTRODUCTION

The discovery of the double-stranded DNA (dsDNA) structures by Watson and Crick more than 70 years ago provided the molecular basis for the stability of DNA and its ability to be replicated and recombined. Furthermore, DNAs are blueprints for living organisms, acting as templates to synthesize mRNA, which is then used to make polypeptide chains that fold into proteins. Replication, transcription, and recombination processes all require duplex DNA to be opened locally so that a single strand can be used as a template. Recent studies have shed light onto how DNA opening is stabilized in replication and transcription and provided some insights into how DNA melting is initiated in replication, recombination, and transcription (*1*–*4*). However, we still only have limited knowledge of the mechanisms behind how DNA melting is precisely initiated and how it is then propagated.

**Fig. 1.**
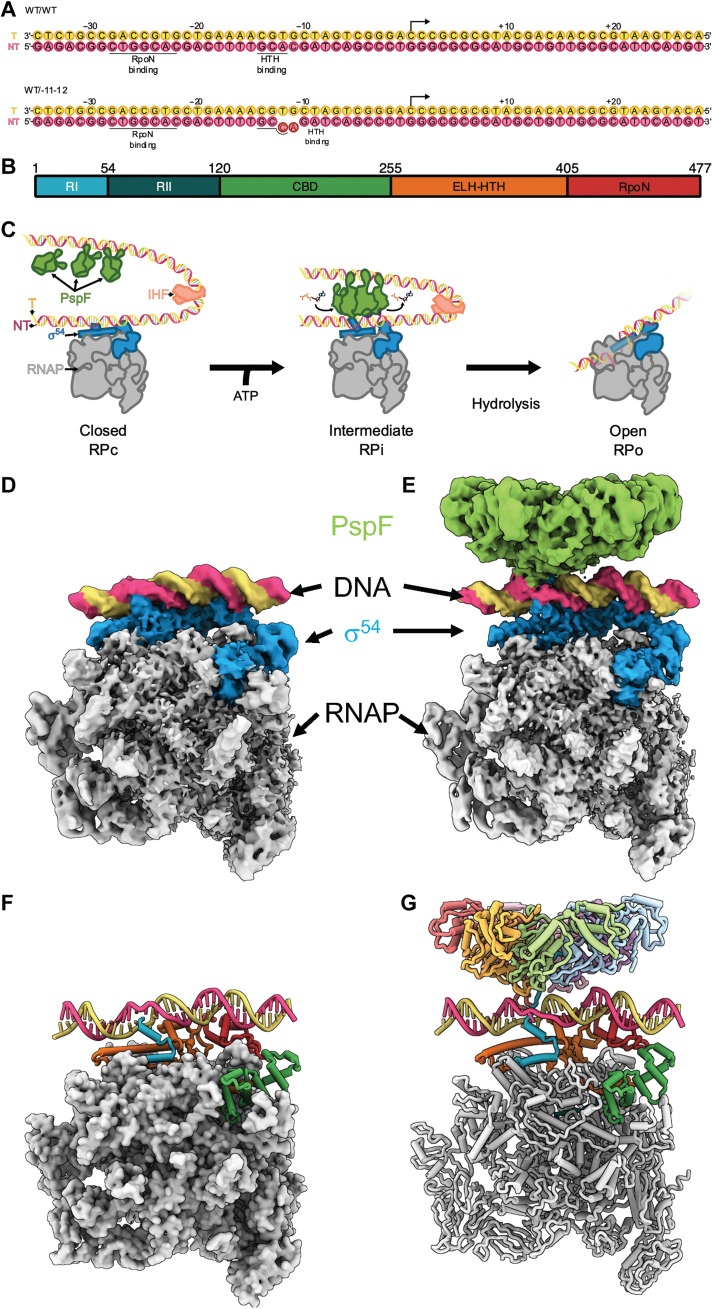
Cryo-EM structures of closed and intermediate complexes of RNAP, σ^54^, and bEBP. (**A**) Promoter DNA used in this study with key regions labeled. (**B**) σ^54^ domain organization. (**C**) Schematics of σ^54^-RNAP open complex formation. (**D** and **E**) Cryo-EM reconstructions of closed complex (D) and intermediate complex (E). (**F** and **G**) Structural models of closed and intermediate complex.

Gene transcription is carried out by the multisubunit RNA polymerase (RNAP), which by itself does not confer DNA sequence specificity. Gene transcription is thus tightly regulated by numerous transcription factors. In bacteria, RNAP relies on dissociable sigma factors (σ) to recognize specific promoters and are thus the primary transcription factors (*5*). Initiation of transcription involves the isomerization process, which converts a closed transcription complex that consists of RNAP, σ, and bound duplex promoter DNA [closed complex (RPc)] to a transcriptionally competent open complex (RPo) where duplex DNA has been locally opened up into a transcription bubble [from −11 to +2 with transcription start site (TSS) being +1] and template strand has been delivered to the RNAP active center (*6*). Substantial advances have been made in understanding the isomerization process, especially how the transcription bubble is stabilized once formed and how transcription bubble is propagated once partially formed (*3*, *4*, *7*–*10*). However, we have limited knowledge on how DNA melting is coordinated with delivering DNA into the RNAP cleft, because of the highly dynamic nature of the process and the likely numerous intermediate states forming. In *Escherichia coli*, seven sigma factors can be broadly divided into two classes based on their sequence homology and regulatory mechanisms (*5*, *11*), of which six belong to the σ^70^ class, represented by the primary sigma factor that controls housekeeping genes. σ^70^ members share conserved domain structures and recognize −35 and −10 promoter DNA sites [35 and 10 base pairs (bp) upstream from TSS]. Most σ^70^-dependent transcription events can occur without activators (*12*). Recent cryo–electron microscopy (cryo-EM) studies of the σ^70^ system reveal a series of intermediate states from RPc to RPo, revealing that the nucleation events of transcription bubble formation involve a series of transient states that could be formed dynamically (*4*). This is consistent with the ability of σ^70^-dependent system to isomerase spontaneously, reflecting a sufficiently low energy barrier to overcome from RPc to RPo. In contrast, σ^54^ recognizes conserved promoter sequences at −24 and −12 (*13*–*16*) (Fig. 1, A and B) and forms stable RPcs that are then fully dependent on specialized activator proteins for transcription (*17*). The activation of RPc requires activators that bind to DNA remotely upstream (−100 to −150 bp) of RPc and use adenosine triphosphate (ATP) hydrolysis to remodel RPc (Fig. 1C) (*6*). σ^54^ controls a diverse range of stress response genes, forms a major variant sigma class of its own, and has wide importance for various bacteria (*18*).

σ^54^ consists of several functional domains and regions (Fig. 1B) (*19*). Biochemical, genetical, and structural studies over the past few decades have revealed that the N-terminal region I (RI; residues 1 to 56) is the main inhibitory element that contributes to the stable closed complex formation (*20*, *21*). It forms an inhibitory structural element with the region III extra-long helix (ELH; residues 314 to 350) and blocks the DNA from entering the RNAP active site as well as constraining the conformational changes in RNAP required for open promoter formation (*21*, *22*). σ^54^ activators contain an adenosine triphosphatase (ATPase) domain and a C-terminal DNA binding domain, which binds to upstream activation sequence, analogous to enhancer-binding proteins in eukaryotes, are thus referred to as bacterial enhancer-binding proteins (bEBPs) (*6*, *18*). bEBPs interact with RNAP-σ^54^ bound at the promoter through its ATPase domain, via DNA looping, often facilitated by DNA-bending protein such as integrative host factor (IHF) (Fig. 1C) (*18*). The ATPase domain of bEBPs belongs to the large AAA+ (ATPase associated with diverse cellular activities) family (*23*). A myriad of cellular processes rely on AAA+ proteins, such as the clamp-loader proliferating cell nuclear antigen (PCNA) and replication factor C (RFC), the replication helicase MCM, ClpX, p97, and proteasome involved in protein unfolding and degradation and dyneins involved in cargo transport (*23*). bEBPs, like most of the AAA+ family of proteins, have been shown to function as hexamers (*24*). Through a combination of structural, biochemical, biophysical, and molecular dynamics studies, an emerging mechanism for a large number of AAA+ proteins is that they act as translocases/unfoldases, with the hexamer enclosing their substrate nucleic acids or polypeptide chains and using ATP hydrolysis within the AAA+ hexamer to translocate/unfold its substrates (*25*).

bEBPs belong to clade 6 of AAA+ proteins and contain a highly conserved signature motif (GAFTGA) within a surface loop/β-hairpin insertion (L1) into the AAA+ domain that is shown to be responsible for engaging and directly interacting with σ^54^ RI (*18*, *24*, *25*). Previous work suggested that ATP hydrolysis of bEBPs would relocate the inhibitory RI, leading to the removal of its inhibitory activity (*21*, *24*, *26*). Our previous work used a promoter DNA with mismatch bases at −12/−11 to mimic the local DNA base unstacking seen in RPc in conformationally sensitive DNA footprinting experiments (*27*). We observe DNA distortions in the form of a widened minor groove downstream of −12, and these appeared to promote transcription bubble formation (*21*, *27*). Because of limited resolution and the lack of clear density for the N terminus of σ^54^, the illusive regulatory N-terminal region has remained a mystery in terms of its precise structures and interactions within RPc and RPo.

In this study, using both fully duplexed DNA and −12/−11 mismatch DNA, we formed transcription complexes, using the AAA+ domain of phage shock protein F (PspF_1–275_), an exemplar bEBP shown to be active in vitro without its cognate DNA binding domain (*28*), ATP hydrolysis transition state analog ADP.AlFx, the RNAP, σ^54^, and a native fully duplexed *nifH* promoter DNA between −35 and +28 (Fig. 1A) (*29*). We formed complexes as previously described and subjected these to cryo-EM analysis (*21*). Biochemical studies showed that under our assembly conditions, a mixture of closed complex (RPc) RNAP-σ^54^-DNA, PspF_1–275_ hexamer, and activator-bound intermediate complex (RPi) PspF_1–275_-RNAP-σ^54^-DNA exists (fig. S1). In the cryo-EM dataset, we observe RPi, RPc, and PspF_1–275_ hexamers. Analyzing these particles separately allowed us to resolve structures of RPc and RPi including promoter DNA from −34 to +2 (Fig. 1, D to G, and Table 1). In regions where the structures are similar, we have used either RPc or RPi reconstruction, whichever has better resolved density, to help in interpreting and building structural models for RNAP, σ^54^, and DNA. These high-resolution structures define the structure of full-length σ^54^ and the nature of DNA bases in the crucial −12/−11 region (arising from both fully duplexed and mismatched DNA sequences), thus revealing the mechanism of initial DNA melting and unveiling multiple interactions made by the regulatory RI N terminus and illuminating how its unusual Q- and L-rich sequence contributes to function. We uncover previously unidentified modes of direct interactions between σ^54^ N terminus and DNA, as well as a mechanism of promoter DNA unwinding facilitated by σ^54^ N terminus and activator interactions (Table 2).

**Table 1.** Cryo-EM data collection and refinement statistics of RPc and RPi complexes obtained from fully duplexed DNA (see figs. S2 and S3). RPc^1^ and RPc^2^ represent the two reconstructions as shown in fig. S2. RPi^1^, RPi^2^, and RPi^3^ represent the three different conformations in the RPi complex as shown in fig. S3. Structural models were obtained on the basis of RPc^1^ and RPc^2^ reconstructions, while the reconstructions from RPi were insufficient to build detailed models.

	WT RPc		WT RPi^*^		
Data collection					
Magnification	81,000		81,000		
Total micrograph	14,780		14,780		
Total frames	40		40		
Total particles	~2.2 M		~2.5 M		
Pixel size (Å)	1.1		1.1		
Defocus range (μm)	−1.4 to −2.6		−1.4 to −2.6		
Voltage (kV)	300		300		
Electron dose (e^−^ Å^−2^)	50		50		
FSC threshold	0.143		0.143		
Detector	K3		K3		
Reconstruction (RELION)	RPc^1^	RPc^2^	WT RPi^1^	WT RPi^2^	WT RPi^3^
Software	Relion	Relion	Relion	Relion	Relion
Particles	31,394	29,321	23,354	21,718	38,490
Symmetry	C1	C1	C1	C1	C1
Resolution (Å)	3.4	3.4	4.3	4.3	3.7
Refinement					
Resolution (Å)	3.4	3.4			
Root mean square deviations					
Bond length (Å)	0.004	0.004			
Bond angle (°)	0.851	0.871			
Ramachandran plot					
Favored regions (%)	92.43	91.76			
Allowed regions (%)	7.55	8.21			
Outlier	0.03	0.03			
Validation					
All-atom clashscore	4.2	4.3			
Rotamer outliers (%)	0.43	0.46			
C-beta deviations	0	0			

**Table 2.** Cryo-EM data collection and refinement statistics of RPi with DNA mismatched at −12/−11. Two regions were further refined to improve quality and resolution. RPi-focus^1^, focus refinement on RNAP-σ^54^-DNA; RPi-focus^2^, focus refinement masked around PspF_1–275_-DNA-part of σ^54^ (see fig. S7).

	RPi^*^	RPi-focus^1^	RPi-focus^2^
Data collection			
Magnification	81,000	81,000	81,000
Total micrograph	10,680	10,680	10,680
Total frames	40	40	40
Total particles	~2.0 M	~2.0 M	~2.0 M
Pixel size (Å)	1.1	1.1	1.1
Defocus range (μm)	−1.4 to −2.6	−1.4 to −2.6	−1.4 to −2.6
Voltage (kV)	300		
Electron dose (e^−^ Å^−2^)	50	50	50
FSC threshold	0.143	0.143	0.143
Detector	K3		
Reconstruction (RELION)			
Software	Relion	Relion	Relion
Particles	33,285	33,285	33,285
Symmetry	C1	C1	C1
Resolution (Å)	3.5	3.2	4.1
Refinement			
Resolution (Å)	3.5		
Root mean square deviations			
Bond length (Å)	0.004		
Bond angle (°)	0.919		
Ramachandran plot			
Favored regions (%)	91.78		
Allowed regions (%)	8.07		
Outlier	0.15		
Validation			
All-atom clashscore	4.7		
Rotamer outliers (%)	0.32		
C-beta deviations	0		

## RESULTS

### Structures of closed complex RPc reveal the origin of DNA distortion and melting

**Fig. 2.**
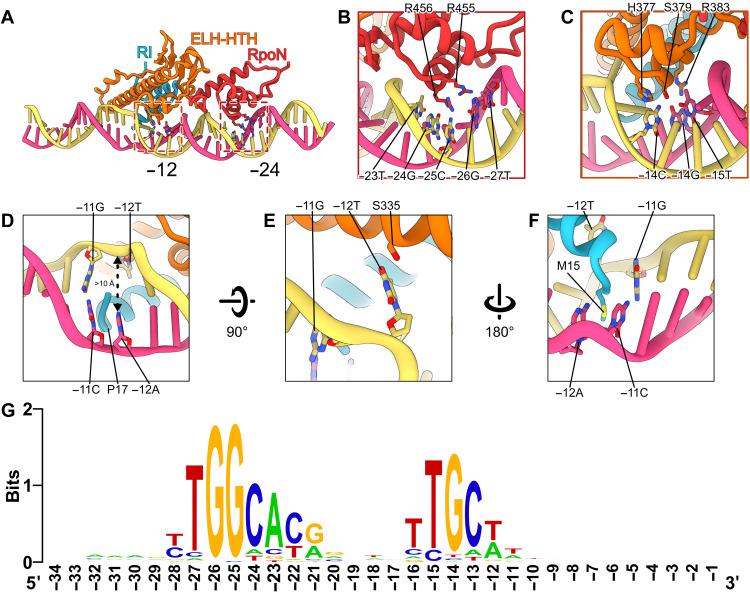
Structure of RPc reveals origins of DNA opening. (**A**) σ^54^ interacts with DNA via several regions. Boxed regions are RPoN and HTH. (**B**) RPoN recognizes specific sequence around −25. (**C**) HTH interacts with −15/−14. (**D**) DNA unwound around the −12 region. (**E** and **F**) Specific interactions to stabilize melted −12 and −11 bases. (**G**) Promoter DNA sequence conservation represented by sequence logo.

For RPc, we observed several structural classes in the dataset and refined two conformations to an overall resolution of 3.4 Å (Table 1 and figs. S2 and S4). The structures are similar except in their relative orientations to RNAP and the degrees of distortions of DNA around −11, suggesting the existence of a range of conformations in RPc with varying DNA orientations. Notably, promoter DNA consensus sequences interact extensively with σ^54^, via ELH-HTH and the RpoN domain as well as RI (Fig. 2A). The invariant R455 and R456 of the RpoN domain recognize the conserved 5′-_−28_CTGGC_−24_-3′ [nontemplate (NT)] sequences at the major groove centered around −25/−24 (Fig. 2, B and G, and figs. S5 and S6). The highly conserved HTH residues (H377, S379, and R383) bind to the adjacent major groove and recognize the conserved 5′-_−14_GC_−13_-3′ (NT) sequences (Fig. 2, C and G, and figs. S5 and S6). HTH and RpoN domain interact extensively, and their expansive interface would impose conformational constrains between the two (fig. S5A), consistent with the strict spacer requirement between −24 and −12 regions in σ^54^ promoters (*15*). The HTH, which is ~31 Å from RpoN domain and thus short of 34 Å between the adjacent major grooves of B-DNA, causes a bend in DNA around −18/−17 toward the RNAP cleft (fig. S5, A and B). Further downstream, RI helix 1 (RI-H1), which forms a structural domain with RIII-ELH, also has limited conformational flexibility. The tip of RI-H1 would clash with the template-strand DNA (T-strand) of a B-form DNA (fig. S5C). Instead, it pushes the T-strand DNA toward the back, resulting in the DNA unwinding around −12/−11 (Fig. 2D and fig. S5C). The unstacked T_−12_ of the T-strand has its base flipped out in both RPc conformations (Fig. 2, D to F; fig. S5D; and movie S1). The NT DNA strand at −12 is in different positions in the RPc structures, suggesting flexibility (fig. S5D). The melted-out bases now create a clear passage measuring >10 Å in diameter in between the DNA strands, sufficiently open to allow a peptide to pass through the promoter DNA (Fig. 2D and fig. S5D).

The flipped-out base of T_−12_ of the T-strand is stabilized by interactions with S335 of σ^54^ RIII-ELH (Fig. 2E). Double mutation of S335A/R336A resulted in significant defects in promoter DNA binding and failed to isomerize to open complex (*30*). Previous studies on R336A showed that mutant holoenzyme has no defects in promoter DNA binding, suggesting that the DNA binding defects observed in the double mutant are largely due to S335A, consistent with the interactions that we observed here (*31*). R336A has been shown to partially remove inhibitions imposed by RI (*31*). R336 interacts with T30 and N337 (fig. S5E). T30 is part of RI-H1, which forms extensive interface with ELH-HTH including L25, L26, L28, V384, and R336 (fig. S5F). Presumably, R336A would disrupt the interactions that are key to hold RI and ELH in position. T30 mutations are defective in transcription including interacting with activators (*32*), and single mutations in L25, L26, or L28 can bypass the requirement of activators (*33*). The structures also explain the σ^54^ promoter sequence preference of 5′-_−18_TTTTGCA_−12_-3′ (NT) (*34*). Four consecutive A-T pairs (from −18 to −15) are energetically favorable for DNA bending around −18/−17 (Fig. 2, A and G). The preferred A-T pair at −12 is also favorable for base melting at position −12 (Fig. 2, A and G).

### Structures of activator-bound intermediate complex reveal N-terminal peptide of σ^54^ threading through DNA strands and are captured by AAA+ activator

In the RPc structure, we could observe density for σ^54^ RI-H1 (residues 15 to 28) with P17 N-capping the RI-H1, causing a sharp turn away from RNAP and into the DNA double helix (Figs. 1F and 2D). M15 was observed near the −11 melted base (Fig. 2F). This raises the possibility that σ^54^ RI N-terminal peptide (residues 1 to 14), although unable to be resolved, probably due to flexibility, could have threaded through the DNA to the opposite side of the DNA from RNAP. It is thus intriguing to see where the σ^54^ RI N-terminal peptide is upon activator binding. Previously, we have shown that activators interact with RI, although it is unclear exactly where it binds because of limited resolution (*21*).

We analyzed the PspF_1–275_-bound RPi complexes and observed a range of conformations, with PspF_1–275_ hexameric ring tilted differently relative to RNAP-σ^54^-DNA (fig. S3). Among the many different conformations/three-dimensional (3D) classes, three conformations are further refined (fig. S3). From the resolution distribution and electron density maps, it is evident that the resolution of PspF_1–275_ and DNA is lower compared to that of RNAP in all three reconstructions (fig. S4), consistent with the structural flexibility between PspF_1–275_, DNA, and RNAP.

Previously, it was shown that DNA with mismatched −12/−11 bases can stabilize closed and intermediate complexes (*21*, *27*). To overcome the conformational flexibility observed in the RPi complex derived from a fully duplexed DNA and to obtain a higher-resolution structure, we subsequently used a DNA substrate with mismatched bases at −12/−11 (mutating the NT strand from A_−12_C_−11_ to C_−12_A_−11_), which we used previously (*21*), and analyzed the structures using cryo-EM (Fig. 1A). As expected, the conformational flexibility observed in this dataset is significantly reduced compared to those using native *nifH* promoter DNA (figs. S3 and S7), and two similar conformations (with modest differences in PspF_1–275_ hexamer ring relative to RNAP) were resolved to an overall resolution of 3.5 and 4.1 Å, with the DNA well resolved from −34 to +2 in both reconstructions (Fig. 1 and figs. S7 to S9). We have thus focused on the higher-resolution reconstruction, which was further refined to 3.2 Å for RNAP-σ^54^-DNA and 4.1 Å for PspF_1–275_ (figs. S7 to S9). The complete molecular models for σ^54^ consisting of residues 1 to 477, the PspF_1–275_ activator of residues 3 to 259, were obtained and helped in interpreting the RPc models above (Fig. 1, D to G).

**Fig. 3.**
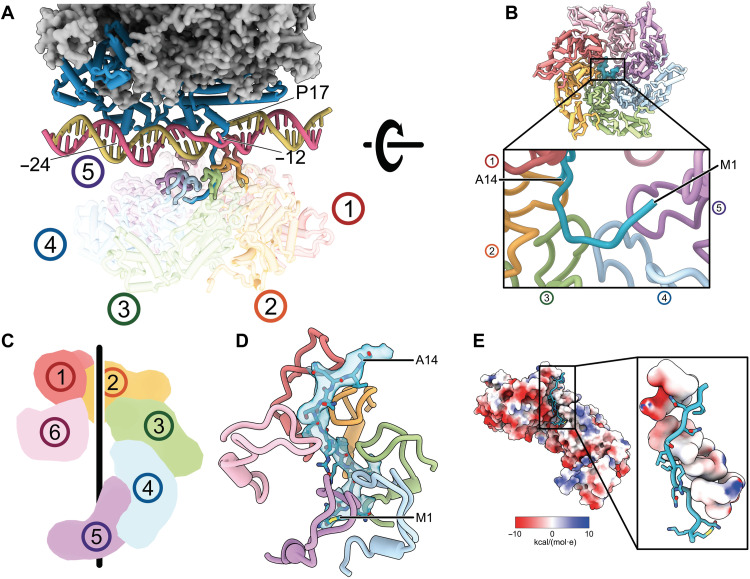
Interactions of PspF and σ^54^. (**A**) N-terminal peptide of σ^54^ enters the PspF hexamer ring once passing in between the DNA strands. (**B**) Five of six PspF protomers engage with σ^54^, and clear density is visible for the σ^54^ N terminus shown in sticks. (**C**) PspF forms a spiral hexamer, with protomer 5 at the lowest point. (**D**) GATGA L1 loops track σ^54^ N terminus. (**E**) σ^54^ N terminus tracked by a largely hydrophobic groove in PspF. Protomers 5 and 6 are hidden for clarity.

**Fig. 4.**
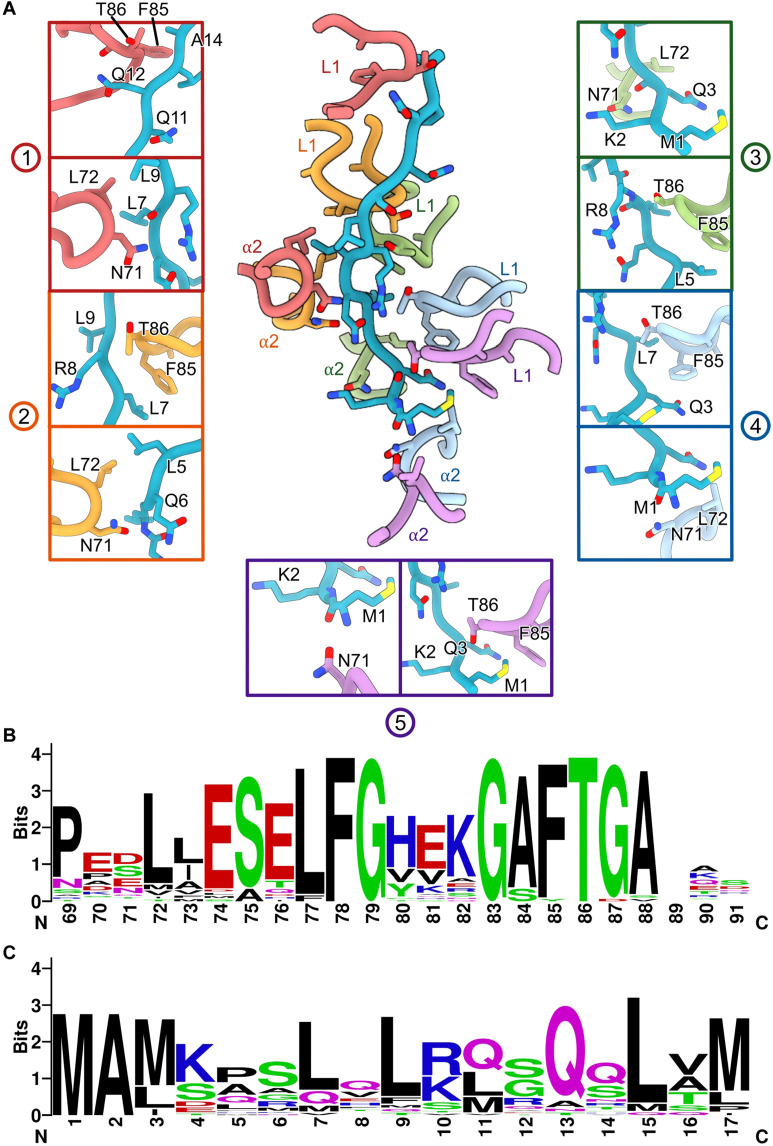
Detailed interactions between the σ^54^ N terminus and PspF protomers. (**A**) Residues 1 to 14 of σ^54^ interact extensively with PspF, especially Phe in the GAFTGA motif interacting with the conserved Leu residues of σ^54^ RI. The circled numbers represent the PspF protomer as in Fig. 3. (**B**) Sequence conservation of L1 loops represented by sequence logo. (**C**) Sequence conservation of RI represented by sequence logo.

The conformation of RPi with −12/−11 mismatched DNA is similar to one conformation (class 1) obtained using the native *nifH* promoter DNA (figs. S3 and S10), confirming that this conformation exists in the native duplex promoter DNA complex, which presumably is stabilized by the mismatched DNA adjacent to the highly conserved GC promoter element (5′-3′) (Fig. 1A). This DNA conformation is also similar to our previous reconstruction (*21*), now with improved resolution, allowing the structures of DNA, σ^54^, and PspF_1–275_ to be resolved. Notably, we observe that the N terminus of σ^54^ RI traverses from the side of RNAP, through the DNA strands, to enter the PspF hexamer pore (Figs. 1G and 3A and movie S2). σ^54^ RI-H1 is N-capped by P17, which also causes the N-terminal fragment to make a sharp turn toward PspF_1–275_ (Fig. 3A). Mutating P17 to Ala abolished the interactions of σ^54^ with activators and its ability to isomerase, in agreement with its important roles revealed here (*30*). Once through the DNA, the σ^54^ RI N-terminal peptide (RI-N; residues 1 to 14) enters the PspF hexamer and interacts with the L1 loops of five of the six PspF protomers (Fig. 3B). PspF forms a nonplanar closed ring (Fig. 3A). When viewed with the AAA+ domain at the bottom while L1 loops on top of the AAA+ hexamer (Fig. 3A), the hexamer forms a right-handed spiral with protomer 5 at the lowest position (Fig. 3C). The L1 loops do not follow a strict spiral arrangement but track the characteristic hydrophobic residues in σ^54^ RI-N, with Phe^85^ (of the GAFTGA motif) in different protomers interacting with conserved Leu residues of σ^54^ RI-N (Figs. 3D and 4A). L1 loops form a hydrophobic channel closely tracking the RI-N (Fig. 3E). The GAFTGA motif in L1 is a signature motif, and mutating Phe to other residues results in defective activators (Fig. 4B) (*35*). Reciprocally, the Leu residues in RI are highly conserved (Fig. 4C) and have been shown to play crucial roles in transcription activation, as mutating pairwise residues resulted in defects in isomerization and transcription (*30*, *36*, *37*).

**Fig. 5.**
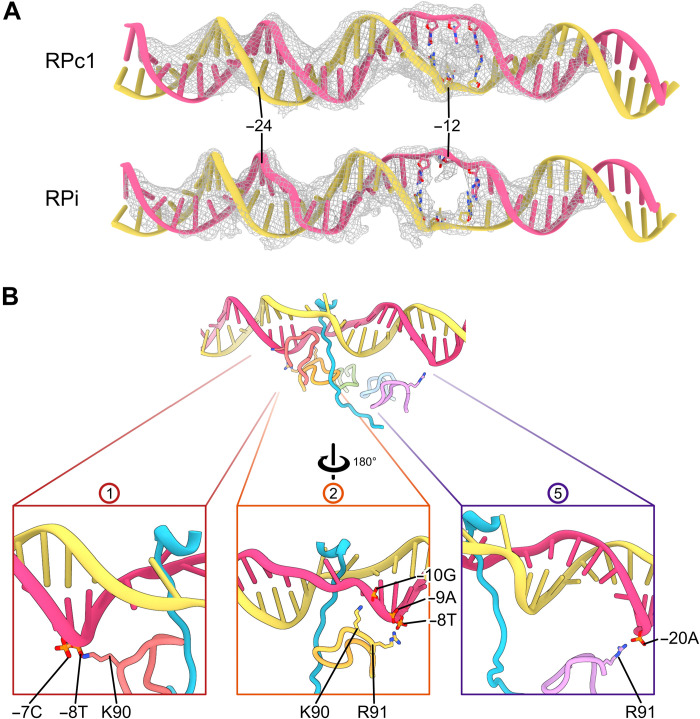
DNA distortions and interactions with PspF in RPi. (**A**) Comparison of DNA at −12 between RPc and RPi; electron density is shown as mesh. (**B**) PspF makes extensive interactions with DNA between −20 and −9, via Arg residues (R90 and R91) of L1 loops with phosphor backbones, L1 loop directly into DNA groove, and next to the σ^54^ RI peptide (insets). Circled numbers indicate the PspF protomers involved as in Fig. 3.

**Fig. 6.**
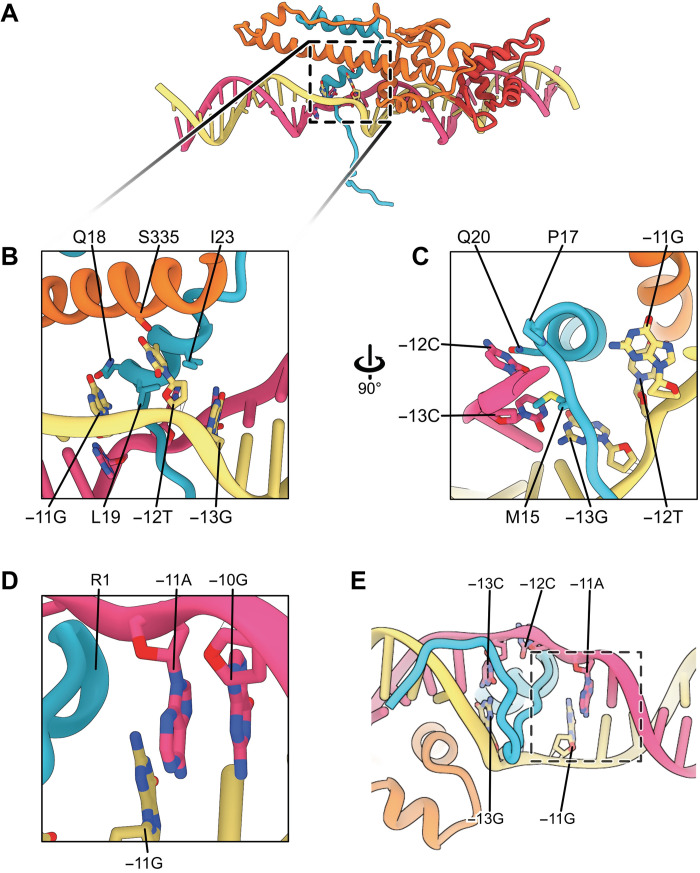
DNA interactions with σ^54^ that stabilize DNA opening. (**A**) RI passes through DNA in RPi. (**B**) Flipped bases at −12 and −11 of T-strand are stabilized by σ^54^ RI and RIII ELH. (**C**) Flipped −12 of NT-strand stabilized by RI. (**D** and **E**) σ^54^ N-terminal peptide passing through DNA, vacated by flipped-out −12 bases.

The local promoter DNA opening at −12/−11, where σ^54^ RI-N passes through, is further enlarged compared to that in RPc (Fig. 5A). The improved quality of the reconstructions allows us to identify detailed interactions that contribute to the DNA opening. The PspF L1 loops interact directly with DNA via phosphate backbones involving several Arg and Lys residues near the GAFTGA motif within the L1 loops (^83^GAFTGAQKR^91^) (Figs. 4B and 5A). One of the L1 loops is positioned in the DNA groove downstream of −12 (Fig. 5B, middle) and directly contributes to the interactions with DNA and the σ^54^ RI sequence that passes through the promoter DNA, thus promoting/stabilizing the further separated strands (Fig. 5B). Similar to those of RPc with fully duplexed DNA, T_−12_ (T-stand) is flipped out and now stabilized by S335 and A22/I23 of σ^54^ (Fig. 6, A and B). The −11 bp are melted with the base of G_−11_ (T-strand) swung toward σ^54^, interacting with the highly conserved Q18 and L19 of RI and K331 of RIII (Fig. 6B and fig. S6). The unpaired NT-strand −12, which was melted in RPc but flexible, is now flipped out and stabilized by Q20 of RI-H1 (Fig. 6C), while the mismatched A_−11_ (NT) maintains base stacking with −10 nucleotides (nt) on one side while with σ^54^ RI on the other side, which now occupies the space vacated by −12 bases (Fig. 6D).

σ^54^ RI peptide makes few specific interactions with DNA when passing through the DNA. However, M15 stacks against the bases of −13 nt, suggesting that hydrophobic residues are preferred in this orientation (Fig. 6, C and E). The stable G-C pair at −13 position might be important to maintain a hydrophobic wall (Fig. 6E), which counterbalances the hydrophilic environment formed by the DNA phosphate backbones, selectively allowing RI, which has multiple rather unusual QL motifs (Fig. 4C), to pass through the DNA opening.

## DISCUSSION

### Origins of DNA melting during transcription initiation in σ^54^-dependent system

DNA opening and transcription bubble formation are key events in transcription. The −11 base flipping and subsequent capture by the σ^70^ W433/W434 dyad are thought to be crucial nucleation events in the σ^70^-dependent system (*3*, *7**)*. Here, we show in σ^54^-dependent transcription that the nucleation of transcription bubble occurs in RPc, due to the interactions between DNA and σ^54^, although activator binding further promotes/stabilizes the −12/−11 DNA distortion. This DNA melting nucleation event involves base flipping and subsequent capture by σ^54^ residues (tip of RI-H1) at −12 position. The changes in −12/−11 DNA structure might correlate with kinetics seen with NtrC at the glnAp2 promoter site where the initial unstable closed complex is transitioned to a more stable closed complex (*38*). Significantly, instead of the side chain of W433 occupying the vacant −11 position as in σ^70^-dependent complexes, the σ^54^ N-terminal peptide (residues 1 to 16) bends 90° relative to RI-H1 and threads through the space vacated by −12 bases. Thus, a prior RPc irreversibly isomerizes from its initial fully base-paired dsDNA state to form a subsequent RPc in which the melted local DNA structure represents the initial transcription bubble formation.

The threading of N-terminal peptide (residues 1 to 14) through DNA is unexpected. Since residue 15 is at the entrance of the DNA opening, the N-terminal peptide can proceed via two routes to go through the DNA opening: (i) The remote N terminus (residue 1) enters the DNA opening first, and subsequent diffusion would help the rest of the peptide through, reminiscent of putting a thread through a needle’s eye, or (ii) residue 14 first enters the opening, followed by residue 13 and subsequently pulling the rest of the peptide through. This would involve a peptide hairpin intermediate in the DNA opening, reminiscent of using a needle threader. The first scenario mainly involves diffusion of a peptide through the DNA opening and thus might be energetically more favorable. However, the initial insertion of the N terminus into the DNA opening could be slow. The >10-Å opening in DNA is sufficient to allow either scenario to occur as a hairpin peptide can be narrowed to ~10 Å. Furthermore, the DNA opening can be further enlarged through thermal fluctuations. We also cannot rule out the possibility that activator binding helps with fully threading the peptide as the peptide is only resolved in the presence of activators. However, the intermediate complex RPi is formed in the presence of ADP.AlFx, and the activator has not undergone ATP hydrolysis, a prerequisite for translocating peptide. Therefore, how exactly the N terminus threads through the DNA remains to be resolved.

Unlike the σ^70^-dependent RPc that converts to RPo readily, the initial −12/−11 DNA opening and the following RPc engagement with the activator in the σ^54^ system are insufficient for RPo formation. Our structures here reveal that despite the initial DNA unwinding/melting, the RI-H1 and ELH remain in similar positions as in RPc, so inhibiting open complex formation. The ATP hydrolysis of PspF will likely result in different interactions with DNA, through which RI is being threaded. Thus the ATP hydrolysis dependent motor functions of the AAA+ protein could directly pry open the two DNA strands of the double helix using the GAFTGA loops to interact and pull apart the strands while RI peptide acts as a “pry bar”.

Our current RPc models obtained from fully duplexed DNA are similar to those we obtained previously with mismatched DNA bases at −12/−11 [Protein Data Bank (PDB) code: 5NSR; fig. S10B] (*21*). We observed clamp opening in our previous RPi structure (PDB code: 5NSS) (*21*, *39*). In the current study, we observe some clamp fluctuations in both RPc and RPi, but the differences in RNAP-σ^54^ conformations are small. Comparisons between our current RPi structure and that of 5NSS reveal different DNA paths and slightly different PspF orientations relative to RNAP (fig. S10C). Given the conformational flexibility that we observe in our current dataset and the limited resolution of the previous reconstruction (overall 5.8 Å with ~10 Å for PspF) (*21*), it is possible that the current RPi structure represents the very initial engagement between PspF and RPc, while the structure of 5NSS represents an assembly of states that are slightly more advanced in the transition from RPc to RPo. The limited resolution in the previous reconstruction prevented us from resolving the N terminus of σ^54^ and the DNA bases in both RPc and RPi (5NSR and 5NSS), so the exact nature of DNA opening is unclear. To understand how ATP hydrolysis of bEBP leads to the release of inhibition by σ^54^ RI, further DNA opening, loading, and eventually open complex formation await further studies.

### bEBPs are multitasking AAA+ proteins

Many AAA+ proteins act as protein and nucleic acid translocases/helicases. Spiral hexamers have been observed in many AAA+ helicases/translocases, and a sequential/hand-over-hand mechanism has been proposed for several of them (*40*–*42*). DNA clamp loaders, such as PCNA or RFC, although forming a spiral, only contain five active ATPases with a further degenerated domain in the sixth position (*43*). These proteins have been shown to transition from an open ring to a closed ring during DNA loading (*44*). It is possible that bEBPs initially exist as a more open ring and closes upon binding to the N terminus, reminiscent of PCNA or RFC loading onto dsDNA, although it is also possible that the N terminus enters the preformed hexamer. For many AAA+ translocases, especially those that are shown to translocate polypeptide chains such as those in ClpXP and proteasome, Vps4, Yme1, and p97/cdc48, for example, use aromatic residues (W, Y, or F) in the pore loops to sandwich peptide side chains (*40*, *42*, *45*–*48*). In these translocases, a pronounced spiral hexamer tracks an extended polypeptide chain with direct interactions with main-chain atoms. In the PspF observed here, it uses L1 loops, which are unique insertions [helix 2 insertion (H2I)] found in bEBPs and other clade 6/7 AAA+ proteins and located above the AAA hexamer ring and the pore loops (Figs. 3 and 4) (*23*). Furthermore, the PspF hexamer does not track the main-chain atoms of σ^54^ RI; instead, it makes residue-specific interactions.

The positions of RI-N in relation to the hexamer suggests that upon ATP hydrolysis, σ^54^ RI-N captured by PspF could be translocated further into PspF, leading to the unfolding of RI-H1 and thus releasing the inhibition RI imposed. Exactly how this was carried out and how many residues in RI-H1 are unfolded and how the reactions are terminated remain to be addressed. In addition to the function as a translocase/unfoldase for R1, PspF also acts on DNA, through its pulling of σ^54^ RI through DNA and its direct interactions with DNA outside of the hexamer pore. ATP hydrolysis of PspF will result in different subunits within the AAA+ hexamer being engaged with DNA and thus could potentially pull apart the double helix further. bEBPs thus act as a multitasking molecular machine with both protein and DNA as substrates.

### Unique mechanism in DNA opening and protein-DNA interactions

DNA opening is an essential step in DNA replication, transcription, and repair. In homology-directed DNA repair (homologous recombination), RecA, DMC1, and RAD51 use a β-hairpin loop to interact with and stretch DNA, inserting a hydrophobic (in RecA) or charged (R and P in DMC1 and RAD51) residue into DNA (*49*–*51*), helping with DNA opening. In replication, AAA+ MCM is involved in DNA melting. Recent structural studies of Cdc45-MCM-GINS (CMG) complex, which is the activated complex where DNA melting occurred, showed that H2I, equivalent of L1 in PspF, contributes to DNA unwinding by maintaining interactions with one DNA strand during conformational changes upon CMG formation (*2*). PspF L1 loop, in addition to its roles in tracking and pulling σ^54^, could also act similarly to MCM and RecA/RAD51, in helping with propagating DNA opening by interacting with DNA and inserting Phe into DNA.

Our work here shows that σ^54^ RI-H1 unwinds DNA, nucleating the DNA strand separation. RI-N then inserts itself through DNA, to commit the DNA opening irreversibly. To our knowledge, this is the first time a protein peptide has been shown to directly traverse through DNA strands to drive DNA opening and to engage with AAA+ proteins. This special feature with σ^54^ RI and PspF in initiating DNA opening thus defines a previously unidentified class of protein-DNA interaction network and suggests the possibility of using protein sequences to drive changes in DNA conformation and to form DNA pores in native and synthetic settings.

## MATERIALS AND METHODS

### Antibiotics used in this study

Antibiotics were used at the following concentrations: ampicillin (100 μg/ml), kanamycin (50 μg/ml), and chloramphenicol (34 μg/ml). *E. coli* strains were grown in LB medium at 37°C.

### Protein purification

*E. coli* RNAP was expressed and purified as described previously (*22*, *52*). *Klebsiella pneumoniae* σ^54^ (pET28b-σ^54^) cells were grown at 37°C until OD_600_ (optical density at 600 nm) reached 0.6 and were induced at 18°C with 1 mM isopropyl-β-d-thiogalactopyranoside (IPTG) overnight. Cells were harvested by centrifugation at 4°C (4000 rpm, 25 min). In general, three chromatography purification steps (Ni-NTA affinity, heparin, and size exclusion chromatography) were used. Cell pellet was resuspended in lysis buffer A [20 mM tris (pH 8.0), 500 mM NaCl, 1 mM tris(2-carboxyethyl)phosphine (TCEP), 10% glycerol, and 20 mM imidazole] and lysed by sonication. The soluble supernatant was collected through centrifugation (18,000 rpm, 45 min, 4°C) and loaded onto a Ni affinity column preequilibrated with lysis buffer A. σ^54^ was eluted out by linear gradient increasing of imidazole concentration with buffer B (lysis buffer A with 500 mM imidazole). The eluted σ^54^ protein was pooled and dialyzed against heparin buffer A [20 mM tris (pH 8), 75 mM NaCl, 1 mM TCEP, and 10% glycerol] overnight. The sample was then loaded onto a heparin column, and the protein was eluted out with linear gradient increasing of NaCl concentration with heparin buffer B (heparin buffer A with 800 mM NaCl). The eluted σ^54^ protein was concentrated and loaded onto a size exclusion column (HiLoad 16/600 Superdex, 200 pg) preequilibrated with buffer 25 mM tris (pH 8.0), 150 mM NaCl, 10% glycerol, and 1 mM TCEP. The purified σ^54^ protein was homogeneous as judged by SDS–polyacrylamide gel electrophoresis (SDS-PAGE) gel. It was finally concentrated to 9.7 mg/ml and stored at −80°C in the freezer in small-volume aliquots before the experiment.

*E. coli* PspF residues 1 to 275 (PspF_1–275_-pET28b) were expressed and purified as described previously (*24*). The quality of the purified components is assessed by SDS-PAGE (fig. S1A).

### RNAP-σ^54^ holoenzyme formation

The σ^54^ holoenzyme was formed by incubating core RNAP with a fourfold molar excess of σ^54^ at 4°C for half an hour. Then, the premixed sample was loaded onto a size exclusion chromatography column (Superose 6 Increase 10/300 column, GE Healthcare) preequilibrated in buffer [10 mM tris-HCl (pH 8.0), 150 mM NaCl, 10 mM MgCl_2_, and 5% glycerol]. The holoenzyme fractions were pooled and concentrated to a final concentration of 21 mg/ml. They were stored in −80°C freezer in small-volume aliquots.

### Promoter DNA

For both RPi datasets, *nifH* σ^54^ promoter DNA, a 63-bp nucleic acid scaffold (−35 to +28 referring to TSS at +1) was used (Fig. 1). For the wild-type (WT) promoter DNA RPi complex, template strand (5′-ACATGAATGCGCAACAGCATGCGCGCCCAGGGCTGATCGTGCAAAAGTCGTGCCAGCCGTCTC-3′) and NT DNA (5′-GAGACGGCTGGCACGACTTTTGCACGATCAGCCCTGGGCGCGCATGCTGTTGCGCATTCATGT-3′) were used. To stabilize the intermediate complex RPi, −12/−11 mismatch promoter DNA to mimic initial fork junction formation was introduced into the NT promoter DNA, with the modified sequence of 5′-GAGACGGCTGGCACGACTTTTGCCAGATCAGCCCTGGGCGCGCATGCTGTTGCGCATTCATGT-3′. The template-strand DNA sequence was the native promoter DNA sequence. Both the WT promoter DNA and −12/−11 mismatch DNA were annealed into double-strand duplex DNA before RPi complex formation.

### RPi formation

For both RPi datasets (WT promoter DNA and −12/−11 mismatch DNA), the RPi was formed by first incubating purified holoenzyme with a 1.2 molar excess of promoter DNA at 4°C for 30 min. The holoenzyme-DNA complex was then incubated with a sevenfold excess of PspF_1–275_ and adenosine diphosphate (ADP; final concentration, 8 mM), NaF (final concentration, 15 mM), and MgCl_2_ (10 mM) at 4°C for 30 min and then at 37°C for 5 min inTris Acetate (STA) buffer [25 mM tris-acetate (pH 8.0), 8 mM Mg-acetate, 10 mM KCl, and 1 mM dithiothreitol]. After that, 2 mM AlCl_3_ was added before a further 15-min incubation at 37°C. The trapped intermediate complex was then buffer-exchanged using Zeba Spin Desalting Columns (7K MWCO, Thermo Fisher Scientific, Zeba) into the final buffer [20 mM tris-HCl (pH 8.0), 150 mM KCl, and 10 mM MgCl_2_] and used for cryo-EM. The complex formation was assessed using native PAGE along with RPc, PspF, and PspF in complex with RNAP-σ^54^ (fig. S1B).

### Electron microscopy

For both WT promoter DNA RPi (WT RPi) and −12/−11 mismatch promoter DNA RPi (Mismatch RPi) datasets, the RPi samples {8 mM 3-[(3-cholamidopropyl)dimethylammonio]-2-hydroxy-1-propanesulfonate [CHAPSO] was added before grid making} were applied at a concentration of ~3.5 mg/ml to R2/2 holey carbon grids (Quantifoil). For the WT RPi sample, 4-μl samples were applied to each grid, which was blotted (blot force, 3; blot time, 2 s) and vitrified using a Vitrobot Mark IV (Thermo Fisher Scientific) at 4°C and 95% humidity. The grids were then flash-frozen in liquid ethane and stored in liquid nitrogen before data collection. For the mismatched RPi sample, the grids were first plasma-cleaned (Harrick plasma cleaner, PDC-32G-2, low power) for 30 s. Samples (4 μl) were applied to each grid, which was blotted (blot force, −7; blot time, 3 s). Both two datasets were collected at London Consortium for high resolution cryoEM (LonCEM) on a Titan Krios operated at 300 keV using a K3 direct electron detector (Gatan) in superresolution mode and a pixel size of 1.1 Å/pixel. The Gatan BioQuantum K3 energy filter is inserted as a standard 20-eV slit width during data collection, which was carried out automatically using EPU software (Thermo Fisher Scientific). For the WT RPi, a total of 14,780 movies were collected with a defocus range of −2.6 to −1.4 μm. Each movie was collected with a 4.1-s exposure time with a total dose of 50 e^−^/Å^2^ fractioned into 40 frames. For the mismatched RPi dataset, a total of 10,680 movies were collected with a defocus range of −2.6 to −1.4 μm and a 4.1-s exposure fractioned into 40 frames with a total dose of 50 e^−^/Å^2^.

### Image processing

Image processing for both datasets is summarized in Extended Data Figs. 1, 5, and 7. In general, both two datasets were processed using similar approaches initially. Frame alignment and dose weighting were carried out by MotionCor2 (*53*), then CTF parameters were estimated using Gctf (*54*), and reference-based particle picking was performed using Gautomatch (https://github.com/JackZhang-Lab/Gautmatch, v0.56_cu8.0). All the other data processing steps were performed in RELION 3.1 (*55*). Particles were extracted into boxes of either 280 × 280 pixels (RPi) or 256 × 256 pixels (RPc) with 4× binned. Initial 2D classification was used to remove junk particles for downstream processing.

For WT RPi, after 2D classification, ~1.6 million particles were selected for 3D classification, using a previously determined RPi cryo-EM reconstruction (Electron Microscopy Data Bank ID: 3696) low pass–filtered to 60 Å as a reference model with C1 symmetry. One class composed of density for all the components (RNAP-σ^54^, DNA, and PspF_1–275_) was selected, reextracted to 2× binned, and refined. A further focused 3D classification (masking around PspF_1–275_-DNA) into eight classes, with high *T* value (*T* = 16) and local angular search, was performed. Two different conformation classes that show clear density for all the components were selected and processed separately. Particles belonging to each class were reextracted and unbinned, CTF-refined, and polished separately. One more round of 3D classification into three classes with local angular search and high *T* value (*T* = 16) was performed on the two classes independently. For one class, after 3D classification, only one class was found to consist of all components with sufficient amount of details, and this class was refined to a final resolution at 4.3 Å. In the other class, 3D classifications revealed two different conformations based on the relative orientation between PspF_1–275_ and RNAP. They were further CTF-refined and postprocessed with final resolutions of 4.5 and 3.7 Å, respectively. The detailed processing flowchart of WT RPi is in Extended Data Fig. 5.

From the WT RPi dataset, we have also captured the RPc complex. The previously published closed complex cryo-EM reconstruction (RPc, EMDB ID: 3695) was projected into 2D classes and used as templates for particle picking using Gautomatch (https://github.com/JackZhang-Lab/Gautmatch, v0.56_cu8.0). Particles were extracted into 256 × 256 box sizes and then 4× binned. The data processing flowchart is shown in Extended Data Fig. 1. After several rounds of 2D classifications to remove junks, ~1.6 M particles were selected for 3D classification using RPc reconstruction (EMDB ID: 3695) low pass–filtered to 60 Å as the reference model. One class that consists of RNAP, σ^54^, and DNA was chosen for further processing. Particles within this class were reextracted to 2× binned, further refined, and subjected to another round of 3D classification to search for six classes with local angular search and high *T* value (*T* = 16). Two classes representing two different conformations were selected and processed separately in the following steps. The particles of each class were reextracted, unbinned, CTF-refined, and polished individually. Each class was carried out in one more round of 3D classification independently to search for three classes by skipping angular alignment with high *T* value (*T* = 16). In each conformation, one major class (>50% particles) with good features was identified and further CTF-refined, achieving final reconstructions of 3.4 Å for both classes.

For the −12/−11 mismatched RPi (mismatched RPi) dataset, detailed process procedures are listed in Extended Data Fig. 7. Frame alignment and dose weighting, CTF value estimation, particle picking, and particle extraction were the same as for WT RPi, as described above. After 2D classifications to get rid of junk particles, ~1.5 M particles were selected for 3D classification with global angular search for five classes, only one class representing the RPi complex was selected, and particles (0.5 M) were reextracted to 2× binned and refined. After refinement, another round of 3D classification into eight classes was carried out by conducting local angular search with high *T* values (*T* = 16). Two classes with good quality of DNA and PspF features that showed similar conformations were combined and refined, and one more round of 3D classification was performed to search for three classes by applying local angular search and high *T* values (*T* = 16). The 3D classification generated two slightly different conformations, with one accounting for 65% of the particles (main class) and the other accounting for 24% of the particles (minor class); the remaining class is of low quality and has been discarded. The particles of the remaining two classes were reextracted, unbinned, Ctf-refined, and polished individually. A further round of focus 3D classification (masking the PspF_1–275_-DNA) to search for three classes was carried out for the main class by skipping the angular alignment with high *T* values (*T* = 16); one major class (consisting of >50% particles) with high-resolution feature was chosen for further CTF refinement with a final resolution of 3.5 Å. To improve the local resolution of PspF_1–275_-DNA and RNAP-σ^54^-DNA, focused refinements were carried out separately by applying a mask around holoenzyme-DNA and PspF_1–275_-DNA-partial σ^54^ individually. The quality of the maps was improved, with holoenzyme-DNA to 3.2 Å and PspF-DNA-partial σ^54^ to 4.1 Å. For the other RPi class, the particles were further CTF-refined and postprocessed, with the final reconstruction of an overall resolution at 4.1 Å. The resolution estimates were carried out inside RELION using the gold-standard Fourier shell correlation (FSC = 0.143) criterion (*56*).

### Model building and refinement

Command scripts for map conversion and jelly body structure refinement in Refmac (*57*) were provided by G. Murshudov (UK Medical Research Council-Laboratory of Molecular Biology, Cambridge). For mismatched RPi model building, the previously reported RPi (PDB code: 5NSS) was initially docked into the map using Chimera (*58*). The coordinates were further manually adjusted and refined to fit into density in Coot (*59*). In the previous RPi structure (PDB code: 5NSS), because of limited resolution, the σ^54^ N-terminal residues and some other regions were unclear. With the higher-resolution electron density/potential maps obtained in this work, combining with the AlphaFold (*60*) predicted model of the σ^54^ structure, a complete σ^54^ structural model (residues 1 to 477) was built in. For WT RPc model building, the cryo-EM structure of the transcription close complex (PDB ID: 5NSR) was docked in (*58*). Coordinates were further manually adjusted to fit into the density in Coot (*59*).

For both RPc and RPi model refinements, jelly body and reciprocal-space refinement in Refmac were first performed to maintain geometric restraints (*57*). Afterward, real_space_refinement was carried out in Phenix (*61*) to correct Ramachandran and rotamer outliers. Last, Phenix was run in atomic displacement parameters (ADP or B-factors) mode to assign atom B-factors and to generate the final model statistics (Table 1). Visualizations were carried out in PyMOL, Chimera (*58*), or ChimeraX (*62*).
