# Supplementary material for: Mechanisms of DNA opening revealed in AAA+ transcription complex structures
Source: Sci Adv. 2022 Dec 21;8(51):eadd3479. doi: 10.1126/sciadv.add3479 (PMC9770992; doi:10.1126/sciadv.add3479)
Supplement: Supplementary file 1 — Figs. S1 to S10 [file sciadv.add3479_sm.pdf]

Science Advances
AAAS

Supplementary Materials for

Mechanisms of DNA opening revealed in AAA+ transcription complex structures

Fuzhou Ye *et al.*

Corresponding author: Xiaodong Zhang, xiaodong.zhang@imperial.ac.uk

Sci. Adv. **8**, eadd3479 (2022)
DOI: 10.1126/sciadv.add3479

The PDF file includes:

Figs. S1 to S10
Legends for movies S1 and S2

Other Supplementary Material for this manuscript includes the following:

Movies S1 and S2

Fig. S1. Sample quality and complex formation. A) SDS PAGE gels of individual components used in the study. **B**) Native PAGE gels of complexes. WT RPc: RNAP-σ^{54} in complex with fully duplexed DNA; WT RPi: RPi (RNAP-σ54, $PspF_{1-275}$ in the presence of Mg. ADP.AlFx) assembled with fully duplexed DNA; PspF: $PspF_{1-275}$; PspF-holoenzyme: $PspF_{1-275}$-RNAP-σ^{54}: mismatched RPi (or RPc): RPi (or RPc) formed with DNA with mismatched bases at -12 and -11 positions

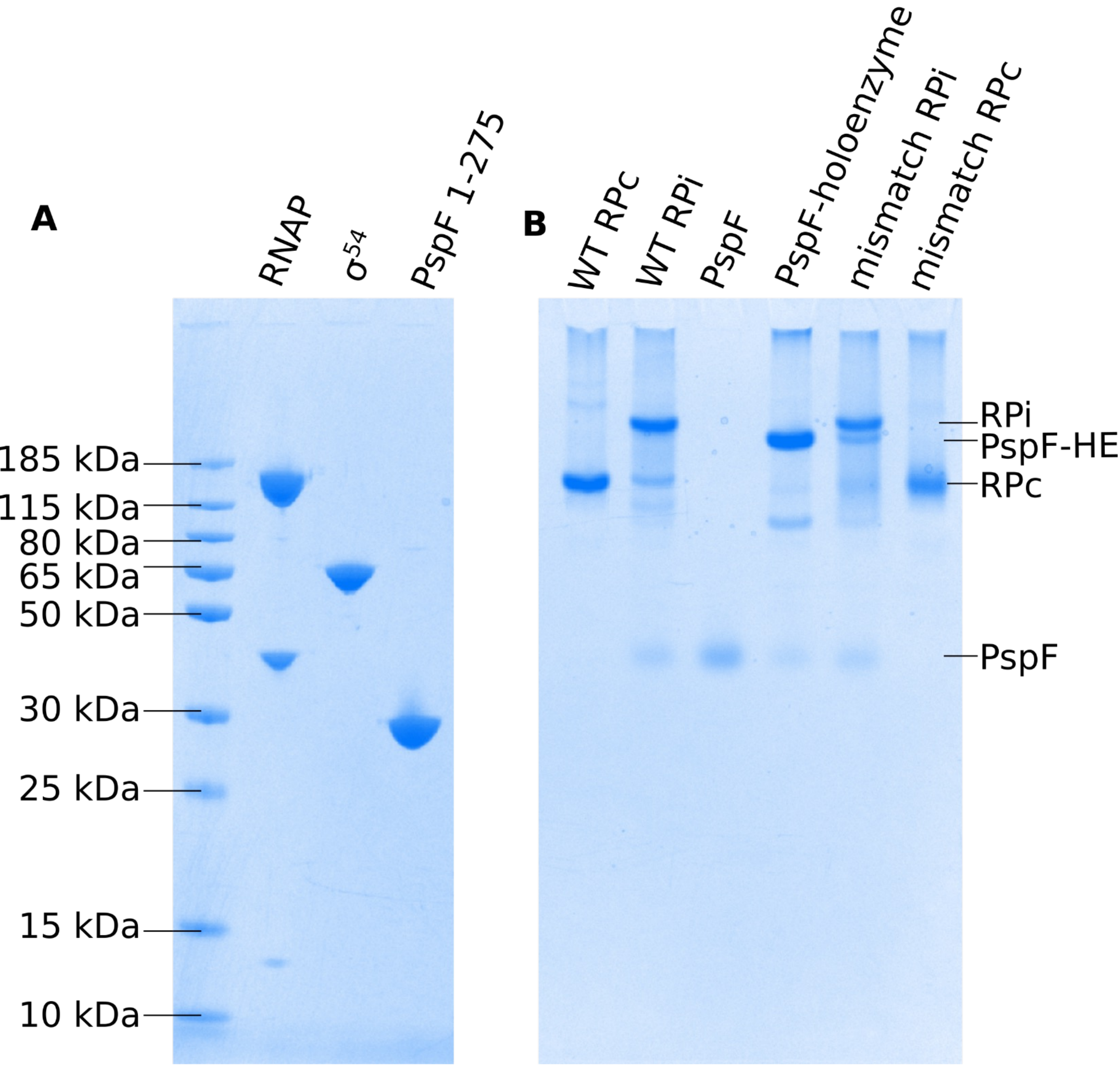

Fig. S2. Data processing flowchart of transcription closed complex (RPc) containing RNAP, σ^{54} and with fully duplex promoter DNA.

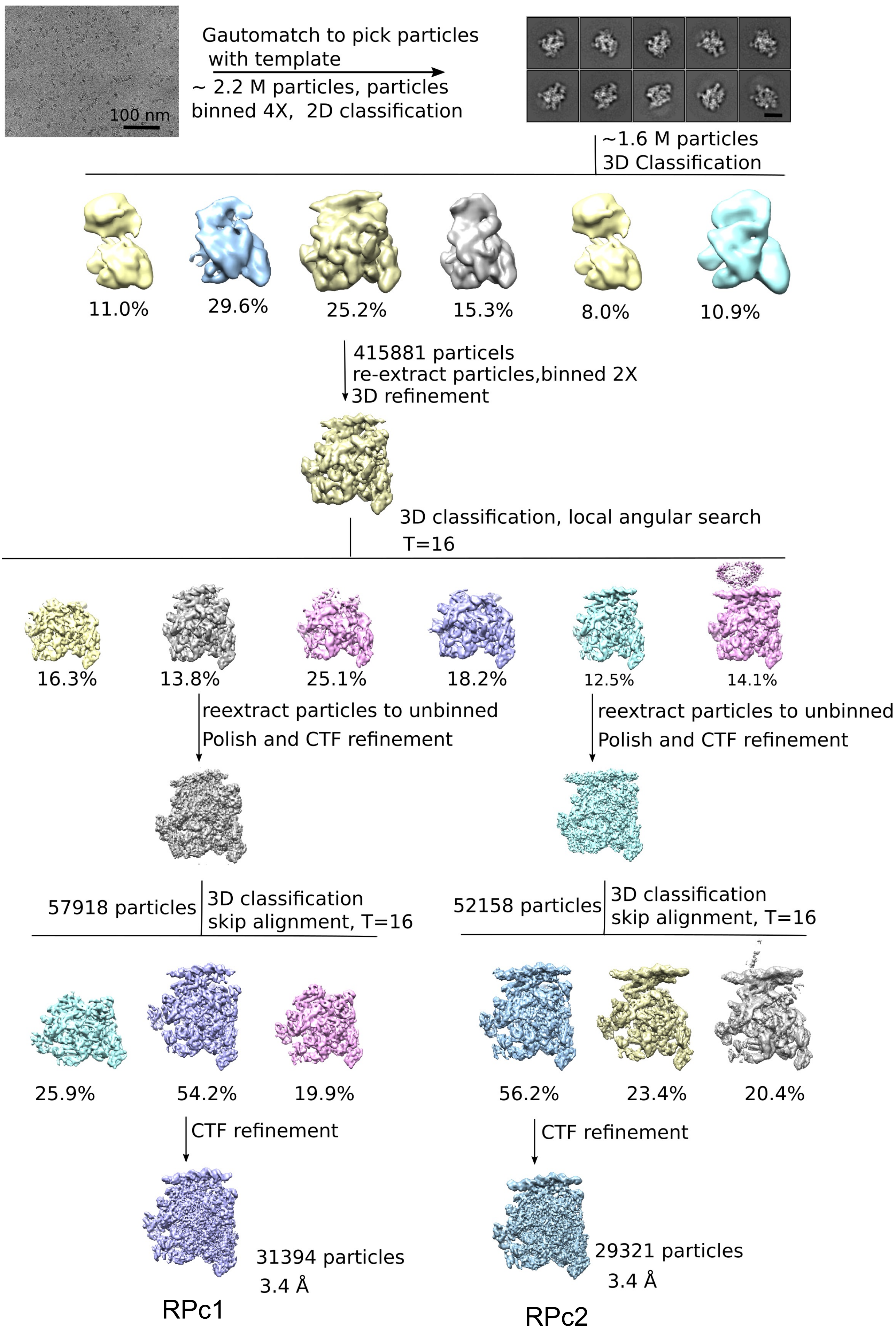

Fig. S3. Data processing flowchart of transcription intermediate complex containing RNAP, σ^{54} , $PspF_{1\text{-}275}$, $ADP.AlF_x$ and with fully duplexed promoter DNA.

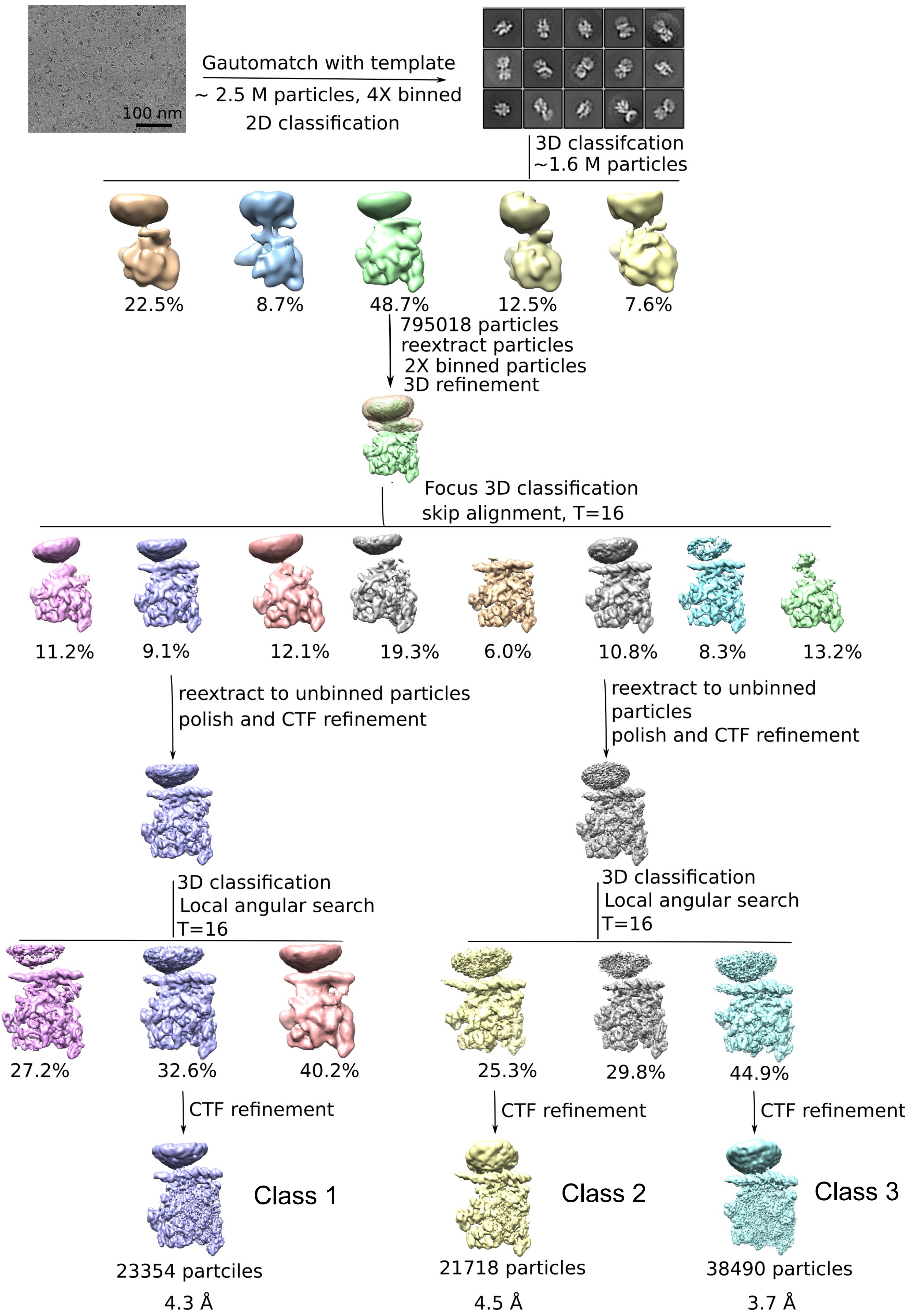

Fig. S4. Resolution maps, angular distributions of contributing particles and FSC curves of RPc and RPi with fully duplexed DNA. **A)**. the two closed complex as in Fig. S2 and **B)**, the three intermediate complex as in Fig. S3. The resolution is determined according to the gold-standard FSC. FSC curves of various maps (corrected, masked, unmasked and phase randomnized) are shown.

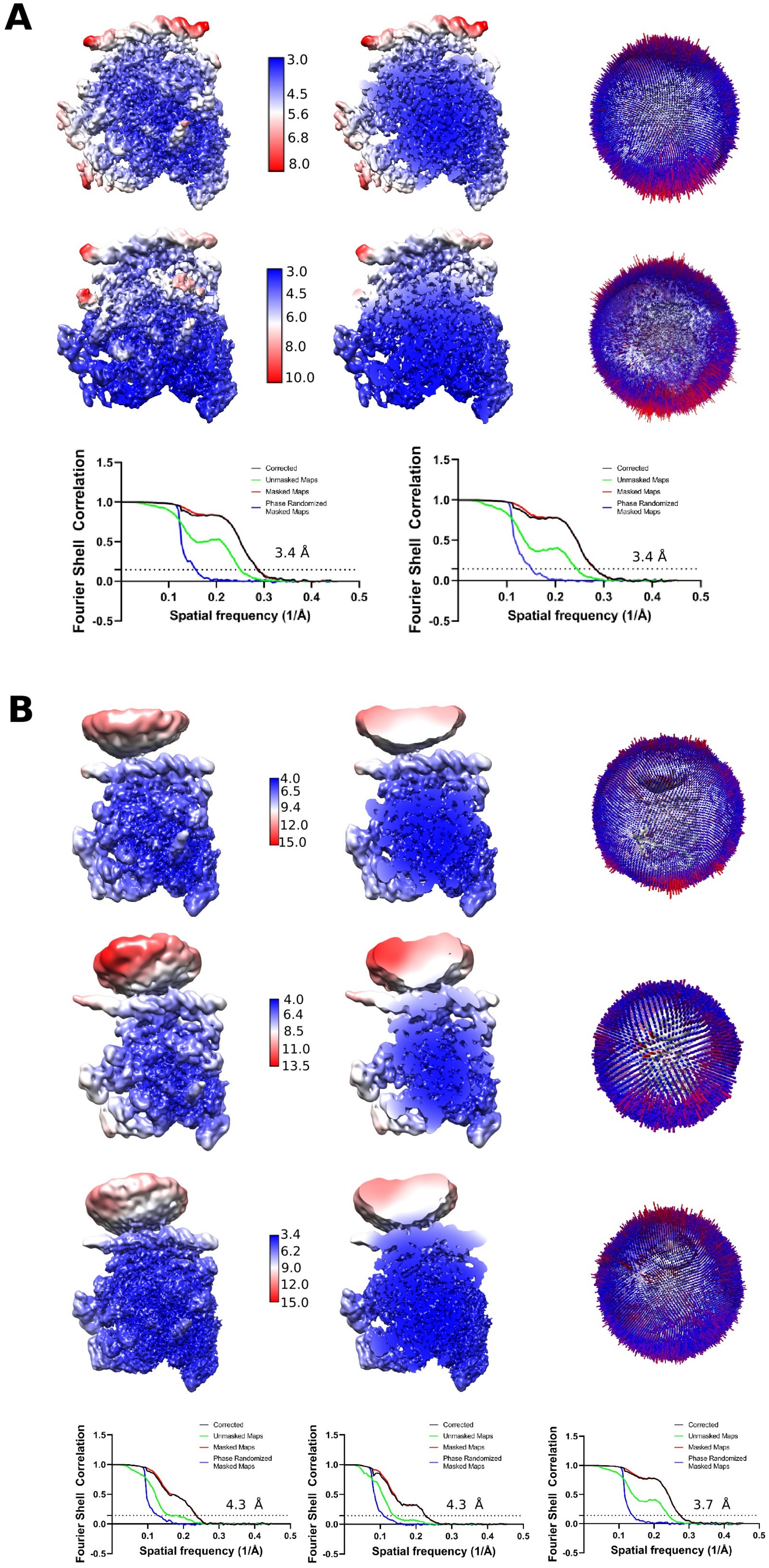

Fig. S5, Distortions of DNA in the closed complexes are caused by σ^{54} interactions, **A)**, DNA is bent and distorted in the closed complex compared to a B-DNA, **B)**, Extensive interactions between RpoN and HTH restrict conformations and cause DNA bending, **C)**, RI-H1 would clash with a B-DNA (grey). Instead it pushes the DNA strand back, causing unwinding in the closed complex (yellow and magenta), **D)**, -12 bases are melted out in both RPc conformations. **E)**, R336 interacts with T30 and N337. T30 is part of R1-H1**, F),** RI-H1 and ELH-HTH interact extensively.

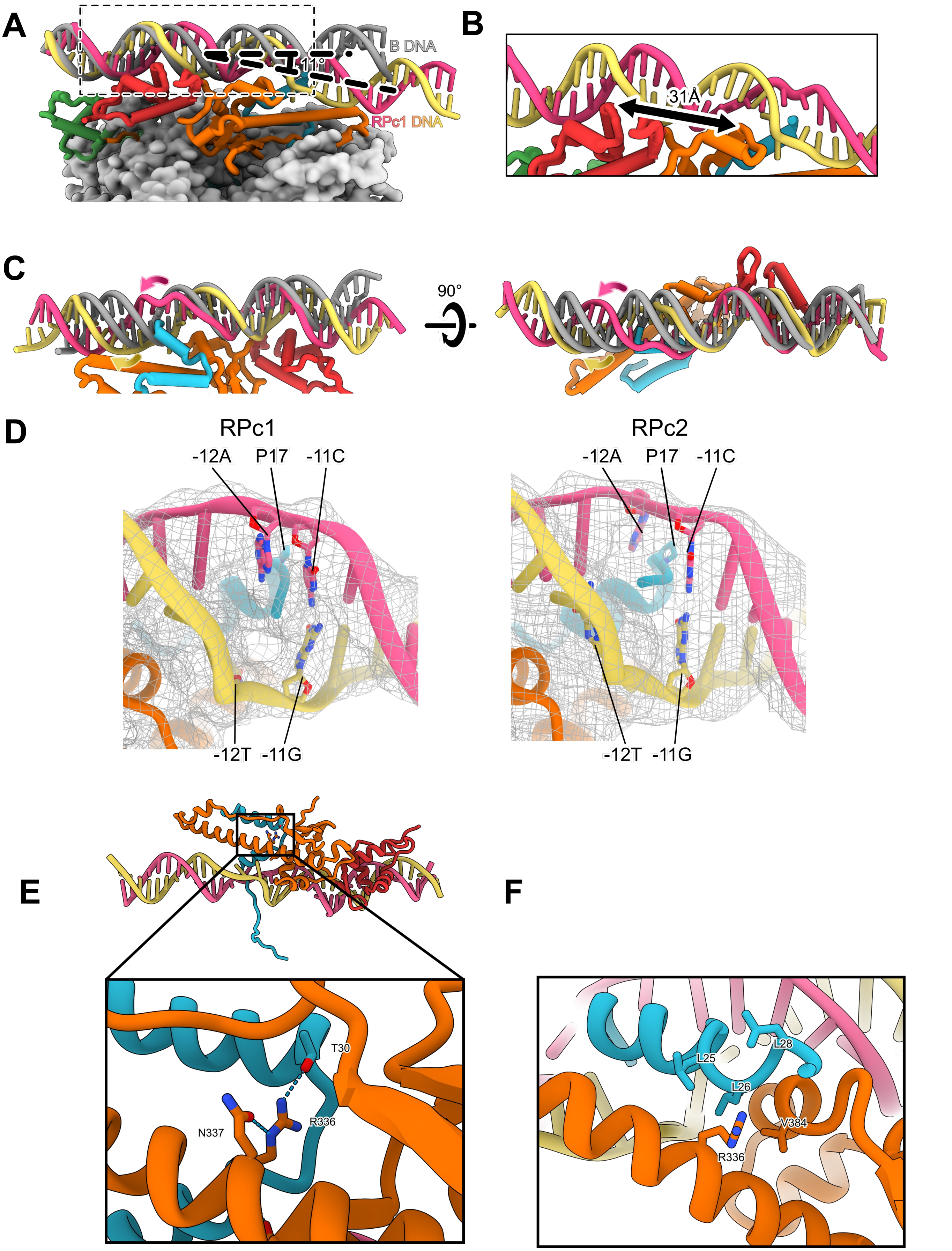

Fig. S6, Sequence conservations of σ^{54} functional regions: A), Region I, **B)**, RIII ELH-HTH and **C**, RPoN domains.

A R1

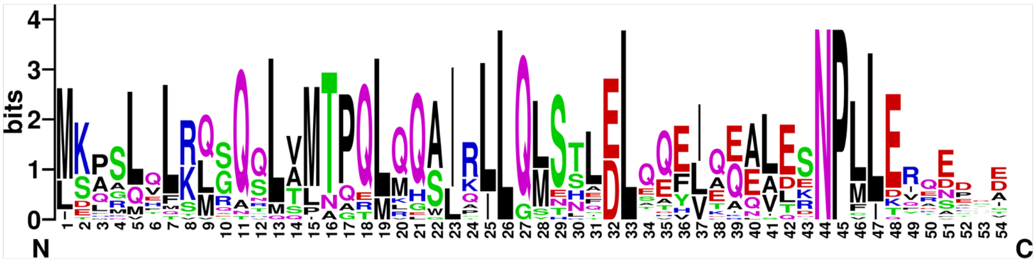

B ELH-HTH

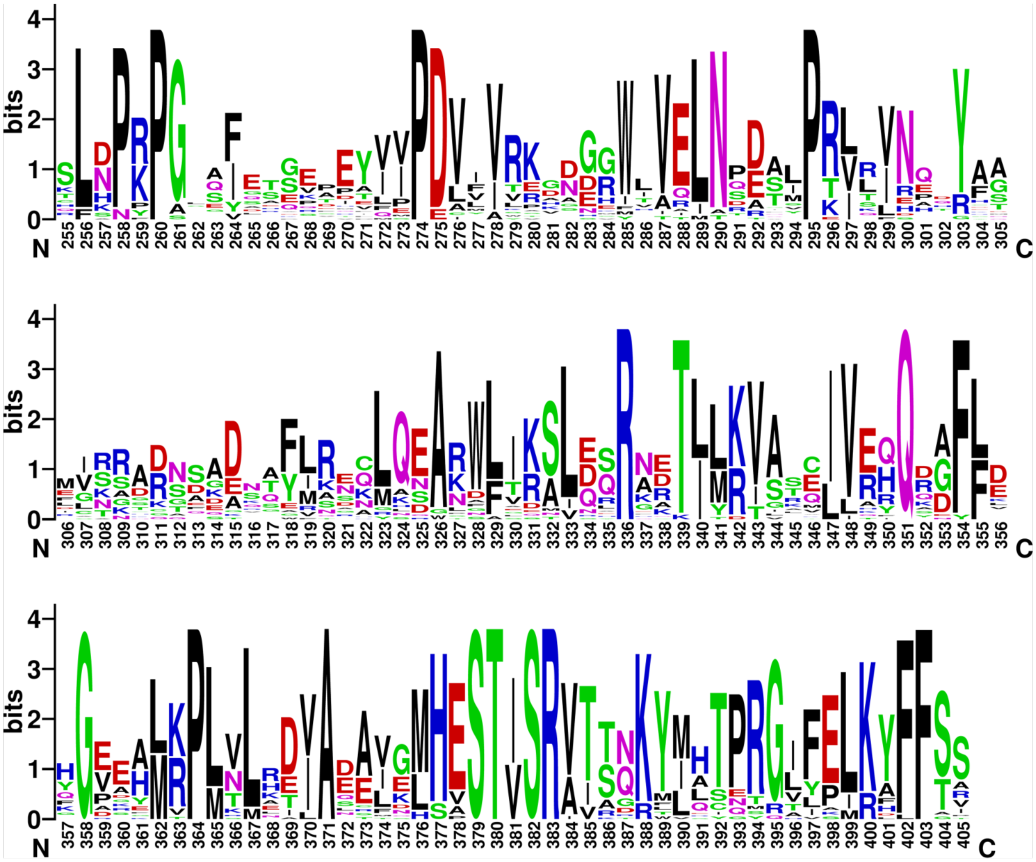

C RpoN

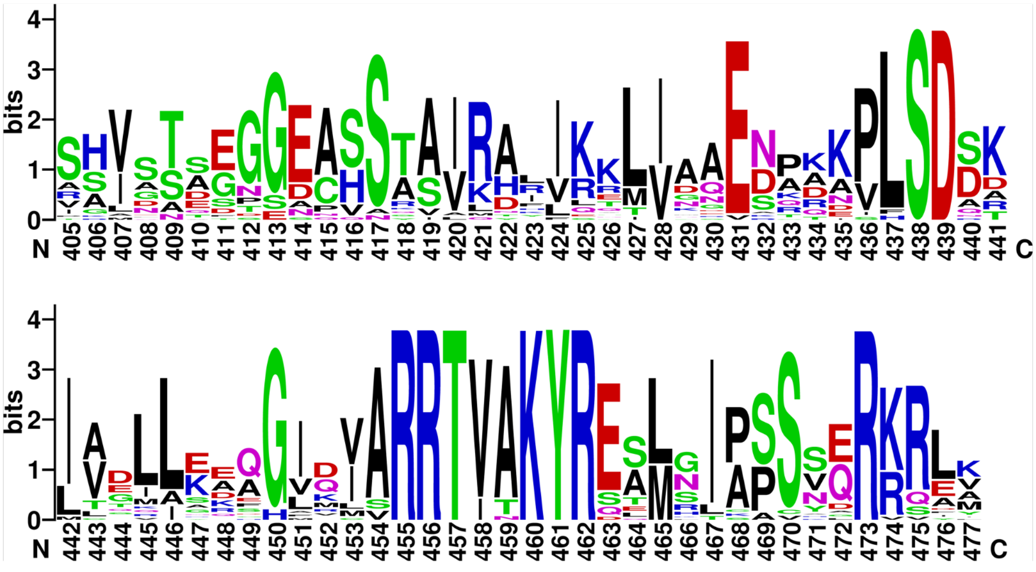

Fig. S7. Data processing flowchart of transcription intermediate complex using mismatch DNA at -12/-11. Yielding reconstructions with an overall resolution of 3.5 Å, and regions PspF1-σ^{54}-DNA achieving an overall 4.1 Å resolution while regions of RNAP-σ^{54}-DNA of 3.2 Å,

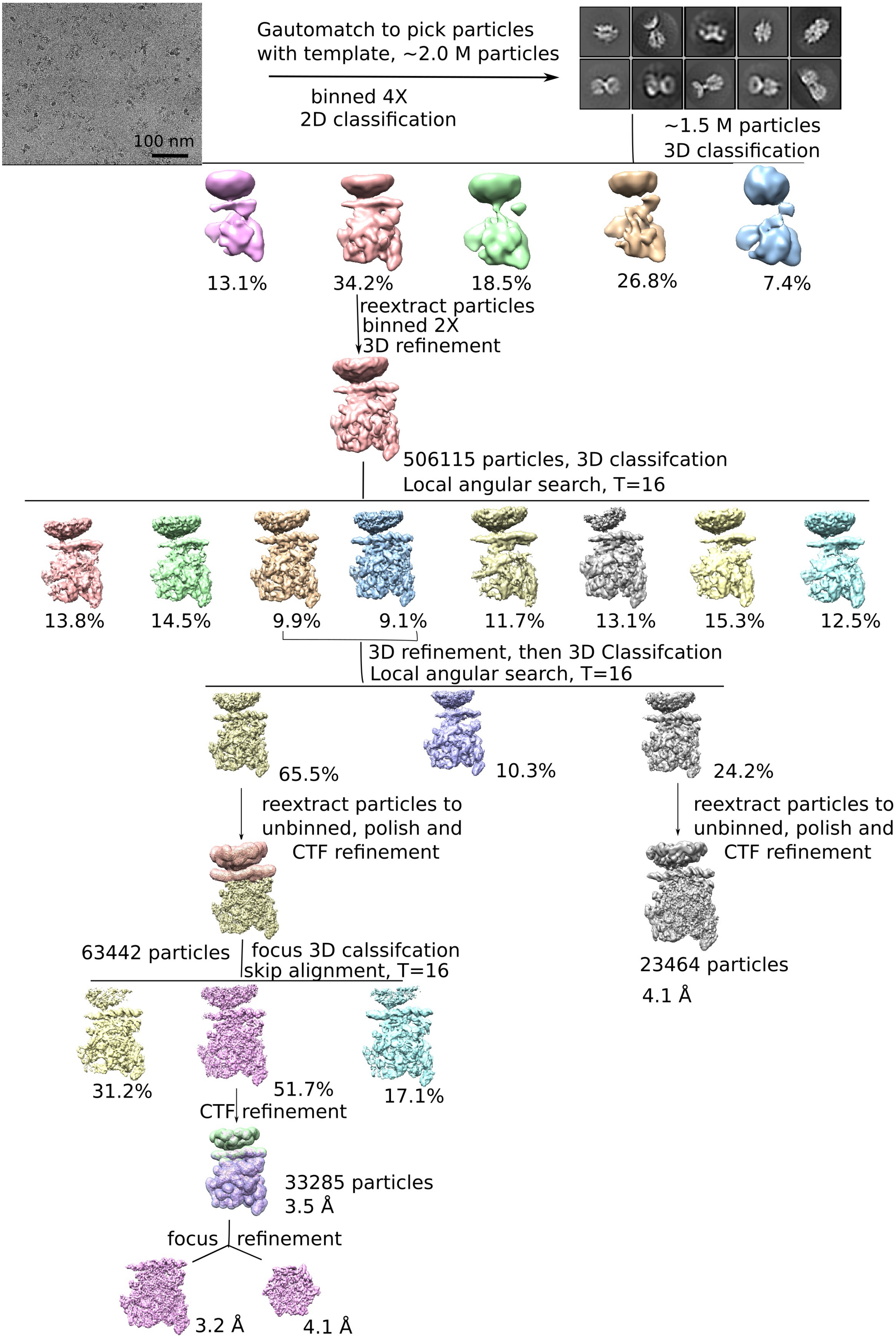

Fig. S8. Resolution maps, angular distributions of contributing particles and FSC curves of two reconstructions of the intermediate complexes with mismatched DNA as in Fig. S7 (A) Also included are the focus-refined maps (**B and C**). The resolution is determined according to the gold-standard FSC.

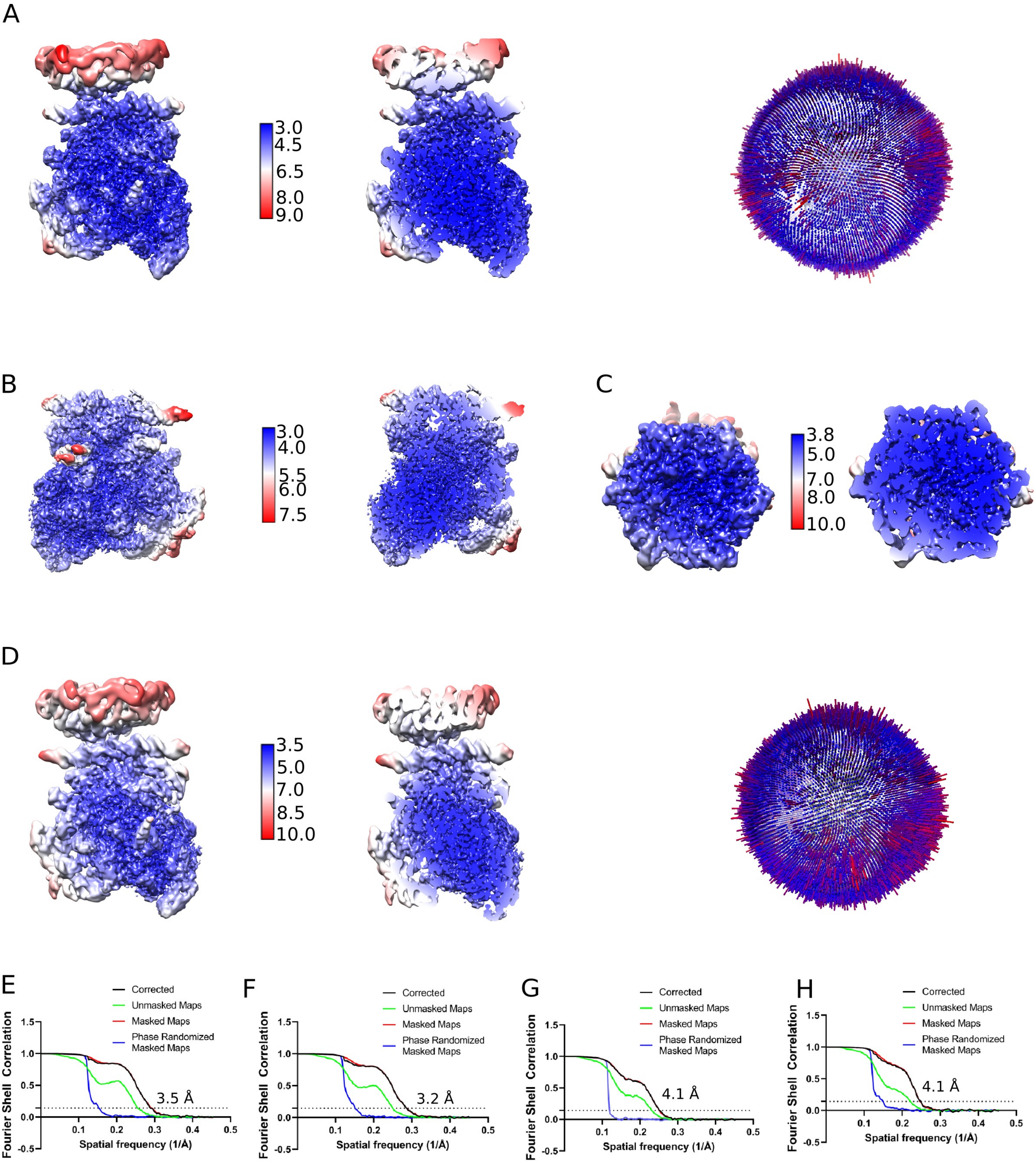

Fig. S9. Representative electron density in different regions of the structures. A). Representative electron density of well-defined regions from the RPi structure containing mismatch promoter DNA. **B**). Representative electron density of well-defined regions in RPc from RPc structures containing fully duplexed wildtype promoter DNA.

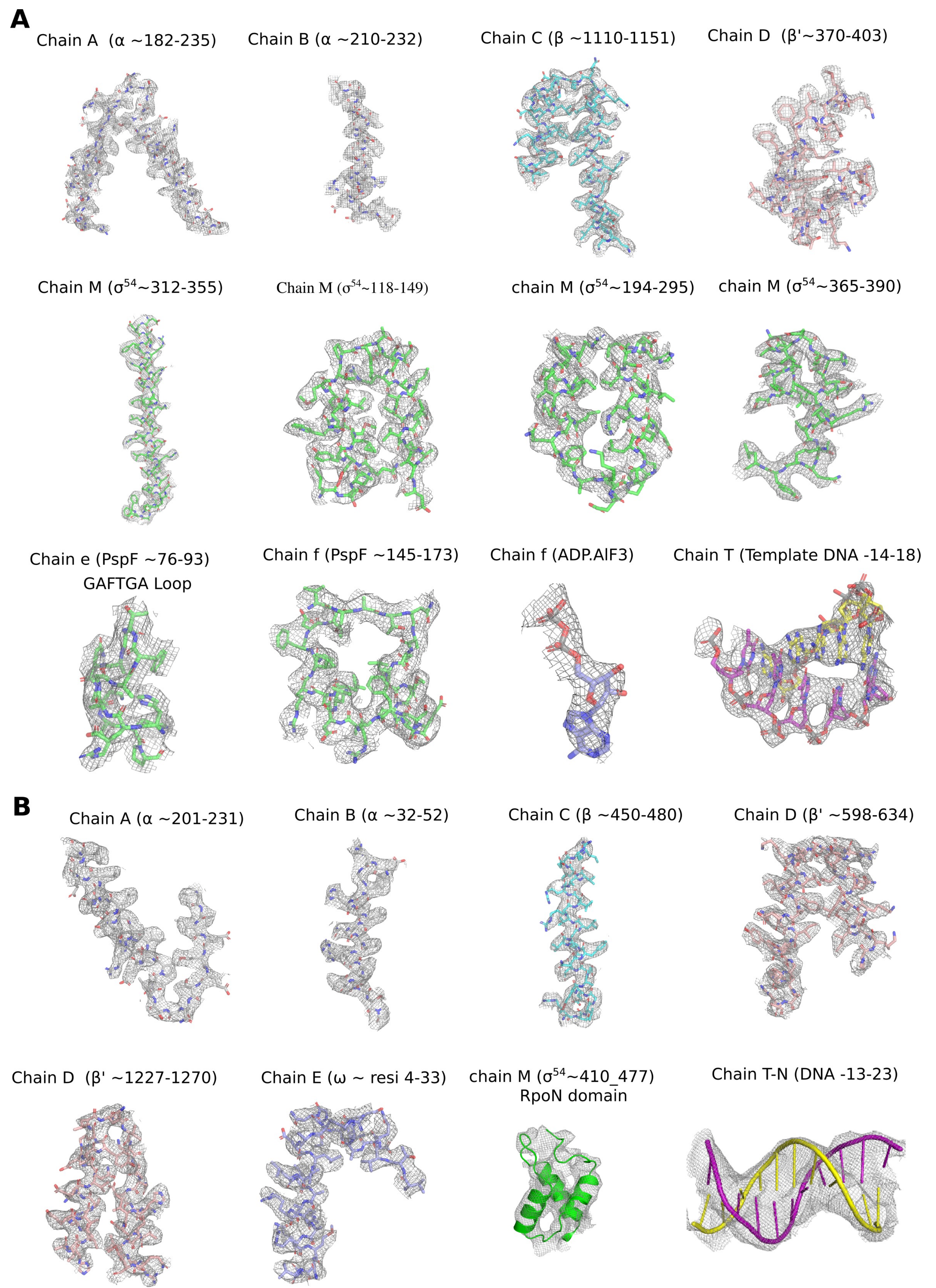

Fig. S10, Structural comparisons of those in this study with those from previous studies. **A).** comparisons of RPi in complex with fully duplexed DNA with those with mismatched DNA at -12/-11. Class 1 as in Fig. S3 is similar to the one obtained with mismatched DNA, both in terms of PspF orientations to RNAP-σ^{54} and DNA distortions. **B)**, Overlays of RPc1 obtained with fully duplexed DNA here (in color) with that previously obtained with DNA mismatched at -12/-11 (5NSR, in grey). **C**), overlays of RPi obtained in this study (color) with that of 5NSS.

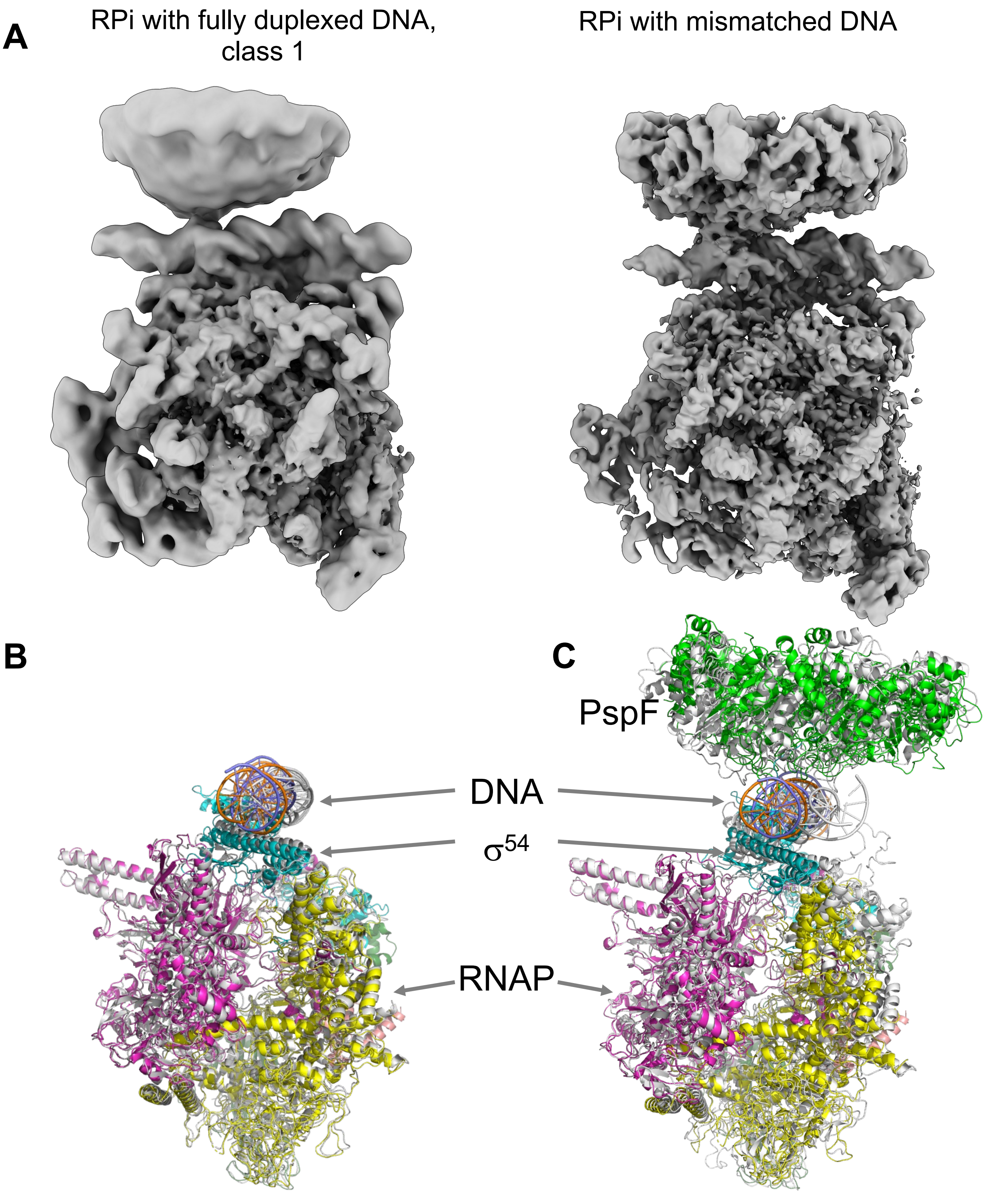

Supplementary Movie 1. **DNA distortions in the closed complexes.** The DNA in the closed complex is significantly distorted compared to a B-DNA, creating an opening of > 10 Å between the DNA strands, sufficient for a peptide to thread through. The distortions are induced and stabilised by interactions with σ^{54}, especially RI-H1 (cyan).

Supplementary Movie 2. **Interactions between σ^{54} RI and the AAA+ activator in the activator-bound transcription intermediate complex**. The N-terminal peptide of σ^{54} (cyan) threads through the DNA strands and entering the AAA+ PspF hexamer. The N-terminal peptide is enclosed by the hexamer.
